# Building responsive governance for learning networks

**DOI:** 10.1002/lrh2.10322

**Published:** 2022-05-26

In Porcaro,^1^ a reference was missing. The reference and where it was discussed is listed below.

“A related analytical frame for determining who ought to be represented in a multi‐stakeholder collaboration is based on influence: the more a stakeholder's power to influence a collaboration, the more essential it is to involve them in the collaboration's governance. Conversely, the more a collaboration's power to influence a stakeholder, the more essential it is to involve that stakeholder in the collaboration's governance.^2^ ^”^
